# Influenza vaccination: key facts for general practitioners in Europe—a synthesis by European experts based on national guidelines and best practices in the United Kingdom and the Netherlands

**DOI:** 10.7573/dic.212293

**Published:** 2016-08-03

**Authors:** George Kassianos, Patricia Blank, Oana Falup-Pecurariu, Ernest Kuchar, Jan Kyncl, Raul Ortiz De Lejarazu, Aneta Nitsch-Osuch, Gerrit A van Essen

**Affiliations:** 1 61, Plough Lane, Wokingham, Berkshire RG40 1RQ, UK;; 2 University of Zurich, Epidemiology, Biostatistics and Prevention Institute, Hirschengraben 84, CH-8001 Zurich;; 3 Department of Pediatrics, Children’s Clinic Hospital, Faculty of Medicine, Transilvania University, Brasov, Romania;; 4 Department of Paediatrics with Clinical Assessment Unit, Medical University of Warsaw, Poland;; 5 Department of Infectious Diseases Epidemiology, National Institute of Public Health, Prague, Czech Republic;; 6 Department of Epidemiology, Third Faculty of Medicine, Charles University in Prague, Czech Republic;; 7 Head of Microbiology and Immunology Service, Hospital Clinico Universitario, Director National Influenza Center, Valladolid, Spain;; 8 Department of Social Medicine and Public Health, Medical University of Warsaw, Poland;; 9 General Practitioner in Amersfoort, The Netherlands

**Keywords:** influenza, vaccination, Europe, guideline, general practitioners

## Abstract

Currently there is no influenza vaccination guidance for European general practitioners. Furthermore, although the European Council recommends a target seasonal influenza vaccination rate of 75% in the elderly (65 years and above) and in anyone aged >6 months with a chronic medical condition, there remain wide discrepancies throughout Europe. A harmonised guideline regarding not only vaccination strategy but also for the consistent diagnosis of influenza across Europe is essential to support a common approach for the implementation of seasonal influenza vaccination across Europe.

This document is based on pre-existing guidelines available in the UK and Netherlands and has been approved by a group of European experts for use throughout Europe. As well as providing a standardised influenza diagnosis, it also reviews the current recommendations for influenza vaccination, the types of vaccine available, the contraindications, vaccine use in special populations (in pregnancy, children, and in those with egg allergy), and concomitant administration with other vaccines. The effectiveness, safety, and timing of the seasonal influenza vaccine are also reviewed. A second section provides practical guidance for general practitioners for the implementation of a seasonal influenza vaccination program, including the selection and notification of those eligible for vaccination, as well as suggestions for the organisation of a vaccination programme. Finally, suggested responses to common patient misconceptions and frequently asked questions are included.

The aim of this article is to harmonise the diagnosis of seasonal influenza and the approach of European general practitioners to seasonal influenza vaccination in order to better identify influenza outbreaks and to move towards reaching the target vaccination rate of 75% throughout Europe.

## 1 Introduction

The European Council currently recommends a target seasonal influenza vaccination rate of 75% in the elderly (aged 65 years and above) and ideally in everyone aged >6 months with chronic medical conditions [1–3]. It has been suggested that this target rate of 75% for populations at risk of complications following influenza infection could be used as an initial milestone towards increasing vaccine coverage rates for all [4,5].

Although certain countries produce and regularly update influenza vaccination guidelines, (e.g., the UK [6–8] and the Netherlands [9]), there is currently no single core document that transcends national European boundaries. Furthermore, there are wide discrepancies in seasonal influenza vaccine uptake throughout Europe, with the UK and the Netherlands being the only European countries that have reached—or are close to reaching—the 75% target vaccination rate in elderly individuals, and vaccination rates in this group are declining in a number of countries, including the European Union (EU) member states [3]. A recent European Commission (EC) report emphasised the need for action at both the EU and at the Member State levels to realise the full gains from seasonal influenza vaccination throughout Europe and recommended the sharing of approaches between member states [3].

Harmonisation of influenza diagnosis would be an important step towards accurate monitoring of epidemiology and identification of outbreaks. Core information on the influenza vaccination itself—including recommendations, special circumstances, contraindications, effectiveness, safety, timing, and vaccine types—should be better communicated to healthcare professionals; this would be facilitated by the harmonisation of guidelines. Additionally, wider distribution of guidelines on the implementation of an influenza immunisation program—including patient eligibility and notification, logistics, record-keeping, and responding to questions that are frequently asked by patients—would improve programme efficiency.

To this end, a group of European influenza experts has combined aspects of the existing influenza vaccination guidelines from the UK [6–8] and the Netherlands [9] to produce this document for use by general practitioners in Europe.

## 2 Background information

### 2.1 Influenza

Seasonal influenza is an acute viral infection of the respiratory tract with a typical incubation time of 1–2 days. It is characterised by fever, chills, headache, muscle and joint pain, fatigue, and sometimes a non-productive cough and can last up to 2 weeks. While for healthy individuals influenza is usually an unpleasant, self-limiting infection, it can be debilitating for 7–10 days and results in significant absenteeism each year. Furthermore, it is very easily transmitted, even by those who have minimal symptoms of illness or are asymptomatic.

Although often incorrectly perceived as not severe or life-threatening, influenza infection can lead to serious complications, particularly in certain populations. These ‘at-risk’ groups include pregnant women, children aged less than 5 years (especially those aged <2 years, an age group in which more hospitalizations occur in previously healthy children, i.e., those without any chronic or underlying disease), the elderly (≥65 years of age), and those with chronic medical conditions, especially those with underlying respiratory conditions or those who are immune compromised [2]. In these groups, infection with the influenza virus can more easily lead to secondary conditions of bacterial origin (including pneumonia, sinusitis, otitis media), hospitalisation, and even death [10].

### 2.2 Epidemiology and burden of disease

There are three types of influenza virus, types A, B, and C, of which types A and B are mostly responsible for seasonal influenza epidemics in humans (type C usually leads to benign upper respiratory tract infection [11]); only type A can cause pandemics.

Influenza type A viruses can be further divided into two subgroups based on two surface antigens—haemagglutinin (H) and neuraminidase (N)—resulting in a classification such as H1N1 or H3N2. Small changes in a sub-type, known as antigenic drift, can occur each year and result in new viral strains and so the necessity for a slightly modified influenza vaccine each year to reflect any changes in the predicted circulating strains (see Section 4.1).

The consequence of mismatch can be lower protection in vaccinated cases, especially those with comorbidities or underlying pathology, and increased mortality in target populations [12].

Recent data suggest that in the European Union 25–100 million individuals of all ages are infected by the influenza virus each year [4,13]. Of these, approximately 38,500 deaths occur each year, mostly (>90%) in the elderly (65 years of age and above) [14]. The mortality, serious complications, school or work absenteeism, and reduced productivity resulting (either directly or indirectly) from influenza lead to an annual economic cost to the EU of €6–14 billion [2,15]. It is undisputed that influenza poses a considerable challenge for healthcare and social care systems around Europe especially during the winter months when case numbers are highest.

## 3 Diagnosis

During the initial contact for influenza-like illness (ILI), the general practitioner determines from the history whether a full physical examination and/or virological confirmation will be necessary. Patient questioning should include the following:

- Duration of symptoms
- Acuteness of symptoms
- Description of symptoms: ask about fever and cold chills, coughing, nasal complaints, sore throat, general malaise, muscle and limb pain, and headache
- Duration and course of fever
- Contact with anyone with ILI
- Is there anyone in close contact (e.g., at home) who may be immunosuppressed?

If the incidence of influenza is low, then a physical examination and/or virological confirmation will be of more positive predictive value than in epidemic situations [16].

Generally, physical examination is important in order to elicit signs and complications of influenza. Virological confirmation is necessary only for a clinically detected outbreak in a care home or other communal living establishment (e.g., university halls of residence, hospital) or when requested by public health authorities, so that prophylactic measures can be introduced [17].

It is worth recommending seeing the patient if comorbidities are present or if one or more of the following are reported (indicating possible pneumonia and an increased risk of complications) [18,19]:

- Shortness of breath
- Expectoration of purulent or sputum
- Raised temperature
- Oxygen desaturation.

The presence of all three of the following criteria can be used to identify ILI, although it should be noted that this definition, while being very specific, is less sensitive and so is a good epidemiological marker but may miss individual cases [20–22].

- An acute onset
- At least one of: fever, malaise, headache, muscle and limb pain
- At least one of: coughing, sore throat, shortness of breath, nasal congestion.

## 4 Influenza vaccination

### 4.1 Recommendations (WHO, EC)

The EC (based on evidence provided by the European Centre for Disease Prevention and Control [ECDC]) [3] and the World Health Organization (WHO) recommend vaccination as the most effective means to prevent seasonal influenza and as the first-line intervention to control the impact of seasonal influenza on public health at a population level.

For the populations at risk of serious complications following influenza infection, a target vaccination coverage of 75% is recommended [2,3].

Influenza vaccination is also recommended for healthcare workers, and it has recently been suggested that the target of 75% could be used as an initial step towards aiming for an ideal of vaccination of all healthcare workers who have no contraindications [4].

As well as the at-risk populations and healthcare workers, influenza vaccination can benefit anyone over the age of 6 months (the lower age for which some influenza vaccines are licensed) and without any contraindication.

Each year the World Health Organisation Global Influenza Surveillance and Response System (WHO GISRS) announces the viral strains to be used in the northern and southern hemisphere seasonal trivalent and quadrivalent influenza vaccines: the viral content needs to be updated regularly to combat the evolution of circulating influenza viruses. Classic seasonal influenza vaccines used currently are trivalent influenza vaccines. In addition to these classic seasonal flu vaccines, quadrivalent vaccines containing both B lineages (rather than just one) as well as two A strains are now becoming available. Quadrivalent vaccines are expected to confer immunity against a wider range of influenza strains and so improve efficacy. Continuous surveillance, virus characterisation, and antigenic cartography are all used to develop the WHO GISRS recommendation 7–8 months in advance of the influenza season to allow time for the formulation, production, and distribution of the vaccines [23,24].

### 4.2 Types of vaccine available

There are essentially two types of seasonal influenza vaccine, summarised below:

- Inactivated influenza vaccines (IIVs)—various presentations are available:
  - Administered by the intramuscular route.
  - Administered by the intradermal route (adults and elderly).
- Live attenuated influenza vaccine administered intranasally (licensed for 2–17 years of age, inclusive, in the UK) (available mainly in the UK).

Inactivated influenza vaccines contain either three (trivalent—intramuscular and intradermal routes) or four (quadrivalent—intramuscular route) influenza virus strains (see Section 4.1), while the live attenuated vaccine is quadrivalent.

The vaccines available in the UK (2015–2016 season) are presented in Table 1. Note that each country will need to check local authority recommendations and indications for the vaccines that are available to them.

### 4.3 Contraindications

For all vaccines^a^ (excluding the live attenuated intranasal influenza vaccine [LAIV] [nasal spray], which is mainly used in the UK: see Appendix 1 for LAIV contraindications), please note the following contraindications:

- Severe egg allergy (only for egg-containing vaccines). Note that vaccines that contain only low levels or no egg protein (with an ovalbumin content <0.12 µg/mL, i.e., <0.06 µg for a 0.5 mL dose) may be administered to individuals with mild to moderate sensitivity to egg protein at the discretion of the physician following a risk–benefit assessment (see Section 4.4.3 and Section 4.2). Patients with severe allergy should ideally be referred to specialist centres for vaccination in accordance with existing national recommendations in the prescribing country and should be observed for a reasonable time post-vaccination (onset of anaphylaxis is typically within minutes but can be unpredictable, and so it is not possible to define a particular time period over which all individuals should be observed [25]). See also Section 4.4.3.
- Allergy to other vaccine component, e.g., preservative, or to a previous dose of the vaccine.
- Age <6 months
- Presence of symptoms of acute illness (in which case vaccination should be postponed until the symptoms have subsided to avoid any possible complications arising from vaccination).

Always refer to existing national recommendations and the summary of product characteristics (SmPC).

### 4.4 Special groups and circumstances

#### 4.4.1 Pregnancy:

Pregnancy does not represent a contraindication to influenza vaccination with inactivated (killed— intramuscular or intradermal injectable) vaccines. The seasonal inactivated influenza vaccine is recommended for pregnant women (regardless of the stage of pregnancy) by WHO, and this group represents the first targeted group for influenza vaccination after the WHO priorities are fulfilled. It is also recommended by the European Council. It has not been shown to be associated with any maternal or foetal safety risk [26].

Pregnant women constitute a population at risk of serious complications—both to the mother and to be baby—if infected [27,28]. This risk can be further elevated during seasons in which the H1N1pdm2009 strain is dominant. Specific risks to the baby include prematurity and low birth weight [29] both of which may be reduced by influenza vaccination during pregnancy [30]. It was also shown that the first trimester maternal influenza exposure is associated with an increased risk of any congenital anomaly [27].

Additionally, since influenza vaccination is not indicated for infants <6 months of age (see Section 4.1) vaccinating pregnant women will confer some protection via maternal antibodies to future newborn infants as well as reducing the risk of infection around the neonate [31–34].

The vaccine should be given in accordance with existing national recommendations in the prescribing country.

#### 4.4.2 Children:

Seasonal influenza vaccination is considered suitable by WHO and by the EU council for children aged 6 months and older with chronic underlying medical conditions (especially respiratory or cardiovascular conditions such as cystic fibrosis, asthma, or heart defects, and also metabolic diseases, including diabetes). Severe complications of influenza infection are most common in children <5 years of age, and in some countries (Austria, Estonia, Finland, Latvia, Malta, Poland, Slovakia, and Slovenia [35]), vaccination is recommended for children from 6 months of age who do not have a contraindication. Generally two vaccinations separated by a 4-week interval are required for children <10 years of age; however, specific recommendations for children vary by country, and the vaccine should always be given in accordance with existing national recommendations.

Vaccination (see Section 4.2 for more detail on vaccine types) can be:

- Intramuscular: for all children aged at least 6 months.
- Nasal spray: for children aged 2–17 years of age, inclusive, including children or adolescents with stable HIV infection receiving antiretroviral therapy or those who are receiving topical corticosteroids, standard-dose inhaled corticosteroids or low-dose systemic corticosteroids or those receiving corticosteroids as replacement therapy (e.g., for adrenal insufficiency) except in the following cases (in Europe this is available mainly in the UK):
  - Patients with a history of severe asthma or active wheezing (e.g., those taking oral steroids at the time of vaccination or those prescribed oral steroids for respiratory disease in the 14 days prior to vaccination).
  - Patients with a history of severe asthma or active wheezing who are currently taking a high-dose inhaled steroid (e.g., Budesonide >800 µg/day or equivalent [e.g. Fluticasone >500 µg/day]).
  - Patients with a history of severe asthma or active wheezing in the 72 hours prior to vaccination or those who have increased their use of bronchodilators during this time. If their condition has not improved after a further 72 hours, these children should be offered an inactivated influenza vaccine to avoid delaying their protection.
  - Any current immunosuppressant medication.
  - Patients currently receiving salicylate therapy (other than for topical treatment of localised conditions) because of the association of Reye’s syndrome with salicylates and wild-type influenza infection.
  - A blocked nose (in which case, if very snuffly on the day of vaccination, nasal spray vaccination should be postponed).

#### 4.4.3 Egg protein allergy:

Many seasonal influenza vaccines are produced using chick embryos and so can contain egg protein; such vaccines are contraindicated in individuals who have severe hypersensitivity (anaphylactic reaction) to egg protein (this generally resolves during childhood and has an estimated incidence of 0.5–2.5% in young children [36,37].

However, seasonal influenza vaccines that are egg-free or with a low ovalbumin content (<0.12 µg/mL, i.e., <0.06 µg for a 0.5 mL dose) are now available, and the latter may be suitable for individuals who have only a mild to moderate allergy.

In children, except for those with severe anaphylaxis to egg which has previously required intensive care, the intranasal vaccine can be used; those with a clinical risk factor contraindicating the use of the nasal spray should be offered an inactivated influenza vaccine with very low ovalbumin content.

Children and adults with a history of severe anaphylaxis to egg should be referred to specialists for vaccination in hospital.

The vaccine should be given in accordance with existing national recommendations in the prescribing country.

#### 4.4.4 Simultaneous administration of influenza vaccination with other vaccines:

Although preferentially administered separately, if necessary, influenza vaccines can be safely administered at the same time as other vaccines.

The vaccine should be given in accordance with existing national recommendations and the SmPC [38].

### 4.5 Effectiveness

Following vaccination, antibodies to the influenza viruses contained in the vaccine are formed after 1 week, with levels peaking after 2 weeks and remaining stable for approximately 24 weeks [39].

Vaccination generally reduces laboratory-confirmed influenza illness by 70–90% in healthy adults^b^, with a reduction of 21–27% in hospital admissions due to influenza in the elderly and a 12–48% reduction in the risk of death [2].

As such, vaccination lowers morbidity resulting from influenza virus infection and also reduces mortality following infection, especially in at-risk populations (young infants, the elderly, those with underlying chronic health conditions, immunosuppressed individuals, and pregnant women).

It should be noted that the effectiveness of the seasonal influenza vaccine can vary by year. For example, the vaccine for the 2014–15 season was less effective than in some previous years due to antigenic drift resulting in a mismatch of the circulating virus strain to that contained in the vaccine. However, even in years of a poor match between the circulating strains and those in the vaccine, being vaccinated remains cost effective and may reduce the clinical and economic burden of influenza infection and severity of related complications by offering direct protection against circulating strains contained in the vaccine and potentially some cross-protection against circulating strains not contained in the vaccine [2,10,39].

### 4.6 Safety and tolerability

Seasonal influenza vaccines have a good safety record that dates back over 50 years and large scale studies have shown no safety concerns [40,41].

The only reproducibly proven side effect of intramuscular or intradermal influenza vaccination compared to placebo is a slightly increased incidence of injection site reactions such as pain, redness, and swelling. Although injection site pain (usually mild in severity) can occur in up to 65% of vaccine recipients, it is usually only mild in severity and usually resolves spontaneously within 1–2 days [42].

Systemic reactions occur to a similar extent following intramuscular or intradermal influenza or placebo vaccination [43].

As for any vaccine, certain vaccine components (e.g., egg protein or preservative) can cause allergic reactions that can range from mild urticaria or angioedema to anaphylaxis [43,44].

Temporally associated cases of Guillain–Barré syndrome after vaccination of inactivated influenza virus have been reported, but safety monitoring over many years has failed to establish a clear link [42,43], and a recent study has shown no association between Guillan–Barré syndrome and influenza vaccination— this study was extensive in its scope, spanning 13 years and including almost 33 million patient-years [45].

The vaccine should be given in accordance with existing national recommendations and the SmPC [38].

### 4.7 Timing

In the northern hemisphere, the influenza season is from approximately October to March/April but can start as early as September and continue until mid-May though it is rare that annual influenza epidemics start before mid-November. Most countries start vaccination in the early autumn, but the exact timing is dependent on national recommendations. However, the earlier it is administered the better (in line with existing national recommendations) with peak influenza infection rates usually occurring from December to February. While one dose of vaccine is expected to result in a protective immune response after 2 weeks that lasts the duration of one season (other than in some children who are naive to influenza vaccination and who may require two doses [see Section 4.4.2]) (see Section 4.5) the response can wane with time; as a result, according to the ECDC, some authorities (which may vary year by year) recommend delaying vaccination campaigns until mid-autumn—the start of vaccination should be in accordance with the availability of vaccine and existing national recommendations [46].

For all children aged 6 months to 8 years (up to 9 years in the UK) who require two doses, (see Section 4.4.2) the timing of the first dose should be according to national recommendations, and the second dose should be given approximately (not less than) 4 weeks later.

The vaccine can be offered through to the end of the influenza season (March–April in the northern hemisphere [be sure to check that the vaccine has not passed its expiry date]).

## 5 Implementation of influenza vaccination

### 5.1 Introduction: importance of general practitioner endorsement

It is generally accepted that the level of awareness of the importance of annual influenza vaccination is less than ideal, and misconceptions are common. For example, Mirza et al. describe the lack of awareness and barriers to vaccination in families of children with chronic conditions [47].

General practitioners are best placed to provide medical advice on vaccination guidelines, according to existing national recommendations, including the benefit/risk assessment for individual patients and patient groups, and to answer specific questions from patients.

The role of the general practitioner and his/her team can include the sending of a written notification and informational material including vaccination consultation hours, the provision and availability of information material in the practice itself, organisation and implementation of the vaccination program, and the repeated offering of vaccination (especially to populations at risk of serious complications of influenza infection).

### 5.2 Selecting individuals eligible for influenza vaccination

Individuals at risk of serious complications are usually prioritised for influenza vaccination in national recommendations. These may vary between countries and include:

- The elderly (65 years of age or above)
- Patients with abnormalities and functional disorders of the respiratory tract and lungs (e.g., cystic fibrosis)
- Patients with a chronic heart function disorder
- Patients with chronic neurological disease such as stroke, transient ischaemic attack (TIA), multiple sclerosis
- Patients with diabetes mellitus
- Patients with severe renal disease
- Patients with severe liver disease
- Patients who have received a bone marrow or solid organ transplant
- Patients who are immunosuppressed because of disease or treatment
- Patients with HIV infection
- Children and adolescents from 6 months to 18 years of age who use salicylates long term.
- Patients with a learning disability in an in-patient facility
- Patients with a decreased resistance to infections, such as those with asplenia or splenic dysfunction, coeliac disease, or sickle cell disease
- Pregnant women
- Patients in long-stay facilities or residential homes
- Patients who are morbidly obese (e.g., body mass index ≥40 kg/m^2^).

Additionally, healthcare workers regularly come into contact with both the influenza virus and with populations who may be at risk of serious complications (as listed above).

It is important, therefore, to promote the vaccination of healthcare workers, not only to protect themselves but also to limit the spread of the virus to patients and thereby reduce the overall burden of disease. This important aspect of influenza vaccination has recently been reviewed [4] and includes staff in general practice surgeries, nursing homes, care homes, and hospitals.

### 5.3 Notifying eligible individuals

The general practitioner should inform all individuals eligible for influenza vaccination (or their parent[s]/guardian[s]) using a personal written notification—examples are included in Appendix 3—and informational material. For care homes, the general practitioner should liaise with the management of the home to organise and coordinate the vaccination of both staff and residents.

It should be noted that personal notification and request to attend for vaccination appears to have the greatest effect on increasing vaccination rates and that a higher level of vaccination reduces the workload in general practice. (It is also noted that the countries that have reimbursement policies for at-risk individuals—this varies widely in Europe—generally have the highest rates of vaccination.) This notification could be by letter, email, or alternatively by telephone call or text message at the discretion of the individual practice. Patients who do not respond or who fail to attend scheduled clinics or appointments should be followed up.

### 5.4 Organisation of vaccination

A well-organised vaccination program including the items listed below will improve vaccine uptake in general practice [48] and if possible should be implemented by the general practitioner (it is acknowledged that the practicalities can vary by country):

- The practice should have a named individual responsible for the influenza vaccination program.
- The practice should have a register that identifies all individuals eligible for influenza vaccination (see Section 5.2).
- The practice should ensure that all eligible individuals (see Section 5.2) are contacted (see Section 5.3) and offered the influenza vaccine at a specific appointment or during an open day that could be held on weekend mornings or weekday evenings.
- The practice should update the registers on an ongoing basis throughout the influenza season, paying particular attention to the inclusion of women who become pregnant and those no longer pregnant during the influenza season.
- The practice should be able to submit accurate data on the number of its patients eligible for influenza vaccination, and the number of patients and healthcare workers vaccinated^c^ to any relevant health authority.
- The practice should ensure that sufficient influenza vaccine is available based on past and planned performance and any expected change in patient demography.
- The practice should follow up on all individuals who do not respond or who fail to attend scheduled clinics or appointments.
- Influenza vaccination should start as described in Section 4.7 (and in accordance with existing national recommendations) to maximise protection in a timely manner.
- The practice should collaborate with midwives to ensure that pregnant women are offered the vaccination andto offer the vaccination to newly pregnant women during the season (if this is a recommended population according to existing national guidelines).
- The practice should collaborate with the management of care/nursing homes and other providers of long-stay residential accommodation to ensure that their residents are offered the vaccination.
- The practice should ensure that house-bound patients are offered the vaccination.
- The practice should have flexible opening hours.

### 5.5 Records

In addition to the register that identifies all individuals eligible for influenza vaccination (see Section 5.2 and Section 5.4), it is important to record those who have been vaccinated as well as those who refuse vaccination. Accurate record-keeping is vital for the following:

- Patient safety
- Audit purposes
- To evaluate the vaccine coverage rate and trends over time
- To correlate vaccine coverage with the incidence rate of influenza
- To ensure that an individual who has already been vaccinated is not re-vaccinated in error
- Budgeting.

Changes to the list of eligible patients should be made to the list on an ongoing basis as new patients are registered with the practice, or existing patients become part of an at-risk group (see Section 5.2). This should be the responsibility of the individual who is responsible for the influenza vaccination program, supported by healthcare worker colleagues (see Section 5.4).

### 5.6 Logistics: storage of influenza vaccines

The optimum storage temperature for influenza vaccines is +5°C, and they should be continuously refrigerated between +2 and +8°C [39]. Interruption of the cold chain can render the vaccine less effective.

For this reason, vaccines are distributed under cold-chain conditions, and it is important that the vaccines are placed in a refrigerator immediately after receipt by the practice.

The individual responsible for the vaccination program therefore should ensure that

- practice staff are available at the time of delivery to transfer the vaccines to a refrigerator that has been validated for this purpose;
- there is enough room in the refrigerator;
- the minimum, maximum, and actual temperatures of the refrigerator are monitored twice per day [49];
- robust storage, waste and disposal procedures are in place.

When placing the vaccines in the refrigerator, care should be taken not to place the packaging next to the cooling components of the refrigerator to avoid the possibility of accidentally freezing the vaccines (this can reduce vaccine efficacy: frozen vaccines must not be used and should be destroyed).

The refrigerator should have air circulating to keep the temperature same at all levels and should have a digital thermometer that can be read without opening the door and a minimum/maximum temperature thermometer. Additionally, there should be a lock on the refrigerator and an alarm and/or temperature-recording mechanism in case the refrigerator breaks down.

The syringes should be kept in the outer carton to protect the vaccines from light.

All vaccines that are unused at the end of the season should be destroyed according to defined procedures; vaccines should never be used once past the expiry date.

### 5.7 Communication with patients

To inform those at risk of serious complications if infected by influenza (see Section 5.2) and who do not respond to a notification, it would be useful to contact the individual by telephone, email, or text message to better understand their reasons for not responding and to better explain the importance of vaccination against influenza. This should be organised by the individual responsible for the vaccination program and could be done by a practice nurse rather than the general practitioner.

Maintaining a list of those who refuse the influenza vaccination can be useful both in terms of monitoring the health of these individuals and the occurrence of any new diseases and also with a view to reminding and discussing the importance of influenza vaccination during their regular consultations with the general practitioner.

A selection of common misconceptions by those refusing influenza vaccination together with suggested explanations as well as a list of frequently asked questions with responses is presented in Appendix 2.

## 6 Overall conclusion/summary

Throughout Europe there exists a wide variation in seasonal influenza vaccine coverage rates for populations at risk of serious complications (for whom the European Council recommends a target vaccination rate of 75% in all member states), for health care workers, and for otherwise healthy individuals.

The UK and the Netherlands have well-developed guidelines that are regularly updated and consequently have good vaccine coverage rates while in many other European countries guidelines are less clear and vaccination rates are declining. This disparity in seasonal influenza vaccine coverage and policy has been highlighted by a recent European Commission report that emphasised the need for action at both the European Union and at the Member State levels to realise the full gains from seasonal influenza vaccination throughout Europe and recommended the sharing of approaches between member states.

Furthermore, there is a need to harmonise influenza diagnosis to ensure the accuracy of the monitoring of virus circulation and to identify outbreaks. Better communication and harmonisation are also needed regarding the core information on influenza vaccination itself. Ideally, guidelines on the implementation of an influenza immunisation program should be more widespread and better harmonised between European Union member states.

Based on these needs, a group of European influenza experts has convened to produce this core document, based on aspects of the existing UK and Netherlands guidelines, to cover aspects of seasonal influenza vaccination for European general practitioners and support a common approach for the implementation of routine seasonal influenza vaccination in Europe.

## Figures and Tables

**Table 1.** Types of seasonal influenza vaccine available in the UK (2015–2016 season).

**Vaccine type**	**Number of strains**	**Brand name**	**Route of administration**	**Age range^a^**					
				**≥6 months**	**2–17 years^a^**	**≥3 years**	**≥5 years**	**≥18 years**	**≥60 years**
Inactivated influenza vaccine	Trivalent	Agrippal	Intramuscular	√	√	√	√	√	√
		Influvac	Intramuscular	√	√	√	√	√	√
		Imuvac	Intramuscular	√	√	√	√	√	√
		Inactivated influenza vaccine (split virion) BP	Intramuscular	√	√	√	√	√	√
		Optaflu	Intramuscular	–	–	–	–	√	√
		Enzira	Intramuscular	–	–		√	√	√
		Intanza	Intradermal	–	–	–	–	–	√

Inactivated influenza vaccine	Quadrivalent	Fluarix Tetra	Intramuscular			√	√	√	√

Live attenuated influenza vaccine	Quadrivalent	Fluenz Tetra	Intranasal	–	√		–	–	–

a Age range 2–17 years, inclusive.

Note: Product availability can vary between countries: each country will need to check local authority recommendations and indications for the vaccines that are available to them and adapt this table according to the manufacturer’s SmPC.

## Footnotes

a Note also the following *precautions* to vaccination: moderate to severe illness with or without fever, Guillain–Barré syndrome within 6 weeks prior to vaccination.

b Note that the seasonal influenza vaccination does not protect against viruses that cause can influenza-like illness, such as rhinoviruses or adenoviruses. The seasonal influenza vaccine only protects against influenza virus strains contained in the vaccine.

c Note that the responsibility for vaccination of healthcare workers may vary by country and could be the responsibility of occupational health doctors rather than the general practitioner.

## Abbreviations:

EC: European Commission
ECDC: European Centre for Disease Prevention and Control
EU: European Union
GISRS: Global Influenza Surveillance and Response System
H: haemagglutinin
HIV: human immunodeficiency virus
IIV: Inactivated influenza vaccine
ILI: influenza-like illness
LAIV: live attenuated intranasal influenza vaccine
N: neuraminidase
SmPC: summary of product characteristics
TIA: transient ischaemic attack
UK: United Kingdom
WHO: World Health Organisation

## Appendix 1 LAIV (nasal spray) contraindications and adverse events

### Contraindications

The contraindications for the LAIV (nasal spray) vaccine are:

- Concomitant use of aspirin-containing medications (salicylate therapy) (due to the association of Reye’s syndrome with salicylates and wild-type influenza infection).
- Pregnant adolescents.
- Anti-virals in the previous 48 hours.
- Hypersensitivity to the active substances, to any of the excipients, to gentamicin, to eggs or to egg proteins, or to a previous dose of this vaccine.
- Clinically immunodeficient patients due to conditions or immunosuppressive therapy, for example, acute and chronic leukaemias, lymphoma, HIV infection without antiretroviral therapy (not contraindicated for use in children or adolescents with stable HIV infection receiving antiretroviral therapy), cellular immune deficiencies, high doses of corticosteroids (prednisolone or its equivalent orally or rectally, at a daily dose of ≥2 mg/kg/day for at least one week, or ≥1 mg/kg/day for one month [prednisolone ≥20 mg/day, or for children <20 kg ≥1 mg/kg taken for ≥1 month]).
- Children with a history of severe asthma or active wheezing (e.g., those taking oral steroids at the time of vaccination or those prescribed oral steroids for respiratory disease in the 14 days prior to vaccination).
- Children with a history of severe asthma or active wheezing who are currently taking a high dose inhaled steroid, for example, Budesonide >800 µg/day or equivalent (e.g., Fluticasone >500 µg/day).
- Children with a history of severe asthma or active wheezing in the 72 hours prior to vaccination or those who have increased their use of bronchodilators during this time. If their condition has not improved after a further 72 hours, these children should be offered an inactivated influenza vaccine to avoid delaying their protection.
- Children who are currently taking any immunosuppressant medication.
- Children who have a blocked nose (in which case, if very snuffly on the day of vaccination, nasal spray vaccination should be postponed).

### Adverse events

The most common adverse events for the LAIV (nasal spray) vaccine are runny nose or nasal congestion [39], which are higher in incidence compared to the intramuscular or intradermal vaccines. Additionally, for the nasal spray vaccine, the incidence of fever is a little higher for children aged 2–6 years but not for older children or adults compared to placebo; also, incidences of headache, sore throat, tiredness, myalgia, cough, chills, and sinusitis are higher than for placebo.

## Appendix 2 Misconceptions and frequently asked questions

### Informing patients and refuting misunderstandings

“Last year I had a flu shot and I got sick anyway, so it does not work”*or* “My husband got the flu right after the shot, I do not need it”.

No vaccine is 100% effective, including the influenza vaccine. A reason for this could be because there are circulating strains of influenza virus that are not contained in the vaccine.

However, the vaccine generally reduces the risk of influenza infection by 70–90% (see Section 4.5) and so will reduce the chances that you will get influenza. Even if you do get influenza, being vaccinated will mean that it will be less severe than if you hadn’t been vaccinated as even if the virus strains in the vaccine are not exactly matched to the circulating strains, there is likely to be a degree of cross-protection and amelioration of symptoms.

The influenza vaccine only protects against influenza. There are other influenza-like illnesses or even the common cold, which you can still catch after being vaccinated and which may resemble influenza.

Also, after being vaccinated, it takes up to 2 weeks for you to be fully protected, and so you may still be susceptible to influenza if you are exposed to the virus during this time. It is for this reason that it is important to be vaccinated as early in the season as possible.

Finally, it is important to re-iterate that an individual cannot develop influenza from having the influenza vaccination.

“I am never sick, so never mind about the flu shot”.

Even for many healthy individuals, influenza can still be a debilitating infection that can last up to 2 weeks and that can infect anyone.

For individuals who are at particular risk of serious complications following influenza infection (see Section 5.2), it can result in hospitalisation or even death. Its potential severity should not be underestimated.

The protection of friends and family is also an important consideration: even if you are healthy and not at particular risk of serious complications, vaccination against influenza will help to limit the spread of the virus. This reduces the likelihood of infecting others with whom you may come into contact and who may be at risk of serious complications (this is why vaccination is recommended for healthcare workers—see Section 5.2).

“I got a flu shot last year, so this year I can skip it”.

The influenza virus is susceptible to regular mutations (see Section 2.2) and as such the vaccine from a previous influenza season may not be protective against the viral strains that may be circulating the following year. It is for this reason that WHO reassesses the vaccine composition each year. Additionally, whilst the seasonal influenza vaccine provides protection throughout the influenza season (see Section 4.5 and Section 4.7), immunity will have waned from one season to the next.

For these reasons a seasonal influenza vaccination is needed each season so that new and appropriate protective antibodies are produced each year.

### Frequently Asked Questions

*When am I most at risk from flu?*

The annual influenza season in the northern hemisphere runs from approximately October to March/April, with the greatest incidence of infection usually being between December and February.

For this reason, and as it may take about 2 weeks after vaccination to be fully protected, you should be vaccinated as soon as possible after the vaccine becomes available (this will be in accordance with existing national recommendations and may be delayed so that you are sure to be protected during the period of greatest incidence of infection).

If the recommended timing is not possible for you, it is recommended to have the vaccine at any time that is convenient during the season as you will still receive protection: it is never too late.

*Can I go to work or school if I have been in contact with somebody who has recently been diagnosed with flu?*

Yes. However, if you are a healthcare worker and develop influenza-like symptoms you should not work with individuals who are at risk of serious complications if infected.

*Does everyone need a flu jab?*

Influenza vaccination can be of benefit to everyone aged over 6 months (the age from which influenza vaccines can be licensed).

However, vaccination is most important in the populations described below (more details are provided in Section 5.2), who are at particular risk of serious complications if infected by the influenza virus:

- Elderly
- Pregnant
- Presence of an underlying chronic health condition (for children aged over 6 months and adults)
- Residential or nursing home resident
- Child or adolescent from the age of 6 months.

Additionally, health and social care workers and anyone in regular contact with the elderly should be vaccinated to limit the spread of the virus to at-risk individuals.

Note that there is some variation between countries, and this should be in accordance with existing national recommendations.

*Why are certain groups targeted for the flu jab?*

Groups of people that are targeted for influenza vaccination are generally those who are at risk of serious complications if infected, including hospitalisation and even death. By vaccinating these people and those who come into regular contact with them (e.g., healthcare workers), complications from influenza are minimised.

*Is my child entitled to the flu vaccine?*

If your child is over 6 months of age and falls into one of the groups that is at greater risk of flu, then it is recommended that he/she is vaccinated and he/she is therefore entitled to the vaccine. There is some variation between countries, and this should be in accordance with existing national recommendations.

*How long will the flu jab protect me for?*

The vaccination will provide protection throughout the influenza season from about 2 weeks after being vaccinated.

*Can I have a flu jab while I’m taking antibiotics?*

Yes, as long as you do not have a fever (in which case influenza vaccination should be delayed until the fever has subsided).

*How long does the flu vaccine take to become effective?*

Approximately 2 weeks after vaccination.

*If I had the flu jab last year, do I need it again now?*

Yes, a new vaccination is needed each season. The circulating viruses may change, so one season’s vaccine may not be protective against viruses that may be circulating the next season, and protection from vaccination is said to last one influenza season only.

*Can the flu jab cause flu?*

No. As either killed or significantly weakened viruses are contained in the vaccine (all vaccines contain killed viruses, except for the nasal spray vaccine which contains significantly weakened virus) the influenza vaccination cannot cause influenza.

You may have some side-effects such as a slight temperature, aching muscles, or soreness at the injection site for a couple of days, but these symptoms are not the norm, and other reactions are rare. Many millions of doses of influenza vaccine are administered every season, and so our knowledge of the risks and benefits of influenza vaccination is very comprehensive and reassuring—and any side-effects are minor in comparison to influenza itself.

*When is the best time to get my flu jab?*

You should try to be vaccinated as soon as the vaccine is available (usually by October in the northern hemisphere): note that vaccination may be delayed in some countries to ensure protection during the peak period of incidence, and this should be done in accordance with existing national recommendations. This will maximise your protection throughout the influenza season.

If you wait until later in the winter, you will increase your chances of being infected.

Even if you have had influenza in a particular season, it would still be of benefit to get vaccinated as it may provide protection against other strains of the virus.

*Is there anyone who cannot have a flu jab?*

Yes. Children under 6 months of age are too young to receive the influenza vaccine, and anyone older who has had a severe allergic reaction (anaphylaxis) to an influenza vaccine in the past or an allergy to any component of the vaccine (such as egg protein or the preservative contained in the vaccine) may not be eligible for vaccination. If any allergies are only mild, it may be possible to be vaccinated—in this case the general practitioner should provide advice on an individual basis.

*Why is it recommended that healthcare workers are vaccinated?*

Vaccination is recommended in healthy healthcare workers, who may not be at particular risk of serious complications if infected themselves but are in regular contact with patients who may be infected (the healthcare worker could become infected) or healthy (the healthcare worker could transmit the influenza infection to patients if he/she is infected). As such, healthcare workers should be vaccinated against influenza to minimise the risk of passing on the infection to more vulnerable patients in their care.

Vaccination of healthcare workers also provides direct protection to the individual thereby avoiding illness when medical services may be under pressure during an influenza epidemic.

*Can I have a flu jab if I’m breastfeeding?*

Yes. There is no contraindication to vaccination of breast-feeding women, and it may even be beneficial for a baby under 6 months of age and therefore too young to be vaccinated.

*Is it OK to have the flu vaccine at any time during pregnancy?*

Yes. There is no contraindication to vaccination of pregnant women. In many countries pregnancy is a defined as a risk for complications of influenza, and therefore vaccination is recommended both for the benefit of the mother and of the foetus/baby. The vaccine can be administered safely at any stage of pregnancy.

## Appendix 3 Influenza vaccination letter for patients (examples)

**Table:**

Dear [Name]: **Annual flu vaccination** Your medical condition or your age suggests that you may be at greater risk of the complications of flu. I would encourage you to have your **free, annual flu vaccination** at the surgery on: [Insert dates—preferably ones that allow for people’s working hours and other demands on their time.] Please phone us now on [phone number] to arrange a time on one of these days. *or* Phone the surgery on [phone number] to arrange a time for your **annual flu vaccination.** Flu vaccination provides the best protection against an unpredictable virus, and it is the best overall way to protect yourself and your family from flu, so please do make an appointment. However, if you decide not to have the vaccination, please let us know so we can enter this on your medical records. Thank you. We look forward to seeing you soon. Yours sincerely [GP name—Suggest GP signs letter] [Position/title]

**Table:**

Dear Sir/Madam: I hereby invite you to come and get the annual flu (influenza) shot at the date and time below. The annual flu shot protects you against the severe consequences of the flu. People who are 65 years of age or older and people of any age who have cardiovascular disease, lung disease, diabetes, kidney disease, or low resistance to infection are particularly at risk of becoming seriously ill from the flu. You belong to one of these groups, and so you are eligible for the shot. You can get your shot at: Date: Time: Place: *Did you receive the invitation for the first time?* *If this is the first time you have received this invitation, the information below is important:* *The flu shot is for people who are at extra risk of becoming severely ill from the flu. If you have had the flu shot, the chance of your getting the flu is smaller. If you get the flu despite having had the flu shot, you will become less seriously ill.* *You must get the shot every year; the best time is between mid-October and mid-November.* *The shot sometimes causes slight pain in your arm for a day, but you do not become sick from it.* You will receive the flu shot free of charge. Please bring this letter with you when you come and get the shot. If you cannot come at the time indicated above, please contact the practice assistant to make another appointment. Your general practitioner

## Appendix 4 Expert Group Members

**Chairman:**

Dr George Kassianos, the UK

**Members:**

Hülya Akan, Turkey
Evis Bagdades, Cyprus
Patricia Blank, Switzerland
Jean-Marie Cohen, France
Oana Falup Pecurariu, Romania
Andrei Galev, Bulgaria
Isme Humolli, Kosovo
István Jankovics, Hungary
Predrag Kon, Serbia
Andreas Kostantopoulos, Greece
Zuzana Kristufkova, Slovakia
Ernest Kuchar, Poland
Jan Kyncl, Czech Republic
Matti Maimets, Estonia
Aukse Mickiene, Lithuania
Aneta Nitsch-Osuch, Poland
Raùl Ortiz De Lejarazu, Spain
Tom Schaberg, Germany
Ted Van Essen, the Netherlands
Dace Zavadska, Latvia
